# Relationship Between State-Level Google Online Search Volume and Cancer Incidence in the United States: Retrospective Study

**DOI:** 10.2196/jmir.8870

**Published:** 2018-01-08

**Authors:** Charles A Phillips, Allison Barz Leahy, Yimei Li, Marilyn M Schapira, L Charles Bailey, Raina M Merchant

**Affiliations:** ^1^ Division of Oncology and Center for Childhood Cancer Research The Children's Hospital of Philadelphia University of Pennsylvania Philadelphia, PA United States; ^2^ Department of Biostatistics, Epidemiology and Informatics University of Pennsylvania Philadelphia, PA United States; ^3^ Department of Pediatrics Perelman School of Medicine University of Pennsylvania Philadelphia, PA United States; ^4^ Department of Internal Medicine Perelman School of Medicine University of Pennsylvania Philadelphia, PA United States; ^5^ Center for Health Equity Research and Promotion Philadelphia Veterans Affairs Medical Center Philadelphia, PA United States; ^6^ Department of Biomedical and Health Informatics The Children’s Hospital of Philadelphia Philadelphia, PA United States; ^7^ Penn Medicine Center for Digital Health University of Pennsylvania Philadelphia, PA United States; ^8^ Department of Emergency Medicine Perelman School of Medicine University of Pennsylvania Philadelphia, PA United States

**Keywords:** Google, cancer, incidence, Internet, infodemiology

## Abstract

**Background:**

In the United States, cancer is common, with high morbidity and mortality; cancer incidence varies between states. Online searches reflect public awareness, which could be driven by the underlying regional cancer epidemiology.

**Objective:**

The objective of our study was to characterize the relationship between cancer incidence and online Google search volumes in the United States for 6 common cancers. A secondary objective was to evaluate the association of search activity with cancer-related public events and celebrity news coverage.

**Methods:**

We performed a population-based, retrospective study of state-level cancer incidence from 2004 through 2013 reported by the Centers for Disease Control and Prevention for breast, prostate, colon, lung, and uterine cancers and leukemia compared to Google Trends (GT) relative search volume (RSV), a metric designed by Google to allow interest in search topics to be compared between regions. Participants included persons in the United States who searched for cancer terms on Google. The primary measures were the correlation between annual state-level cancer incidence and RSV as determined by Spearman correlation and linear regression with RSV and year as independent variables and cancer incidence as the dependent variable. Temporal associations between search activity and events raising public awareness such as cancer awareness months and cancer-related celebrity news were described.

**Results:**

At the state level, RSV was significantly correlated to incidence for breast (r=.18, *P*=.001), prostate (r=–.27, *P*<.001), lung (r=.33, *P*<.001), and uterine cancers (r=.39, *P*<.001) and leukemia (r=.13, *P*=.003) but not colon cancer (r=–.02, *P*=.66). After adjusting for time, state-level RSV was positively correlated to cancer incidence for all cancers: breast (*P*<.001, 95% CI 0.06 to 0.19), prostate (*P*=.38, 95% CI –0.08 to 0.22), lung (*P*<.001, 95% CI 0.33 to 0.46), colon (*P*<.001, 95% CI 0.11 to 0.17), and uterine cancers (*P*<.001, 95% CI 0.07 to 0.12) and leukemia (*P*<.001, 95% CI 0.01 to 0.03). Temporal associations in GT were noted with breast cancer awareness month but not with other cancer awareness months and celebrity events.

**Conclusions:**

Cancer incidence is correlated with online search volume at the state level. Search patterns were temporally associated with cancer awareness months and celebrity announcements. Online searches reflect public awareness. Advancing understanding of online search patterns could augment traditional epidemiologic surveillance, provide opportunities for targeted patient engagement, and allow public information campaigns to be evaluated in ways previously unable to be measured.

## Introduction

Cancer is extremely common in the United States with over 1.5 million new diagnoses annually [1]. The 5 most common cancers in the United States are breast, prostate, colon, lung, and uterine [1]. The incidence for some of these cancers changes over time and varies between states [2,3]. Traditional epidemiologic methods from the Centers for Disease Control and Prevention (CDC) and National Cancer Institute (NCI) Surveillance, Epidemiology, and End Results (SEER) Program have a 2- to 4-year delay until incidence data are publicly reported [1]. While we acknowledge alternative cancer surveillance methods cannot replace traditional cancer surveillance and reporting methods, they have potential value if they are able to augment these gold standard methods in real time and offer information on public awareness for important cancer topics.

Approximately half of Americans report searching for cancer and health information online [4,5], and patients with cancer are increasingly seeking information on the Internet [6]. In addition to patients themselves, friends and family members are known to look up health information online for others [7]. Internet search data including Google Trends (GT) have been used to examine public interest in multiple health topics [8-14]. Google Flu Tracker (GFT), a program incorporating Google Correlate data but not GT, is perhaps the most prominent example of work comparing disease incidence to Google search data. In recent years, multiple studies have shown GFT can be inaccurate for a number of reasons including changes in underlying search rates, news coverage, changes in flu season severity, and errors in the algorithm itself [15-17]. The limitations with GFT must be considered in the design of any research focused on search data. In oncology, GT data have been used to examine multiple topics including seasonality of cancer interest [18], interest in cancer screening [19], efficacy of awareness campaigns [20,21], and effect of news coverage [22]. These events have been shown to drive cancer-related Internet activity and could serve to confound the relationship between cancer incidence and Internet search activity. One prior report demonstrated a correlation between Google search volume and cancer incidence and mortality [23] and did not adjust for changes in incidence over time or compare with drivers of online activity.

This study seeks to compare cancer incidence over time for 6 common cancers, as reported by surveillance registries, to GT data. This work is guided by the conceptual model that people with cancer and those in their immediate social networks are likely to use Google to seek information about cancer symptoms, diagnosis, therapies, side effects, and expected outcomes. Therefore, we hypothesized Google searches are reflective of state incidence patterns. Furthermore, we sought to evaluate previously reported sources of influence in Google data that could explain variability in our data.

## Methods

### Data Sources

This research involved free, publicly available, deidentified, online information from the CDC and GT for the years 2004 through 2013 and was deemed exempt from review by the Children’s Hospital of Philadelphia institutional review board.

#### Cancer Incidence

National- and state-level annual cancer incidence was obtained from the CDC’s website for each year of the study period for the 5 most common cancers in the United States, breast, prostate, lung and bronchus, colon and rectum, and corpus and uterine cancers, as well as for leukemia [2]. Leukemia was included as an example of a cancer that is present in both children and adults and could theoretically have a different search pattern compared to the solid tumors. Approximately 10% of leukemia cases are in children and adolescents compared to less than 1% of cases for the other cancers studied [2]. Cancer incidence data were collected for all 50 states and the District of Columbia except Nevada which did not have its incidence listed by the CDC for all study years. For secondary analysis, states and the District of Columbia were ranked by their cancer incidence from 1 to 50 for each cancer of interest.

#### Google Trends

Started in 2004, GT [24] is a free, publicly available, Internet-based application that allows the relative search frequency of different search terms or keywords to be compared to one another over time. It provides longitudinal data from 2004 through the present with the option to provide search data for specific geographic regions such as states or cities. GT presents search volume for a given term as a relative search volume (RSV) with a value between 0 and 100, with 100 being set as the most searched term in a given time period (weeks, months, or years) and other time periods assigned a proportionally lower number. For example, an RSV of 50 indicates half as many searches were performed in that time period compared to the time period with the highest volume of searches where RSV=100. An RSV of 0 indicates no searches were performed. RSV can either track relative interest in a region compared to itself or between that region and other regions. As detailed in the Statistical Analysis section, we used RSV to compare variation over time both within a state and between states. GT adjusts the RSV results for population size; results from populated areas are comparable to less populated areas.

We selected the search terms used in GT *a priori* using layman’s terms for the common cancers. Our GT search terms were: “breast cancer,” “prostate cancer,” “colon cancer,” “lung cancer,” “uterine cancer,” and “leukemia.” Cancer search terms were entered into GT in 2 ways. First, for the primary analysis, each of the 6 cancer search terms was used individually to obtain the annual RSV for each state from 2004 through 2013. For example, if Kentucky were the state that searched for “breast cancer” the most in a given year, it would have a value of 100, while Kansas would have a value of 50 if it searched for “breast cancer” half as often as Kentucky in that year. Second, all cancer search terms were compared relative to each other for the United States as a whole for the study period, 2004 through 2013, to contextualize national trends for cancer type over time.

The secondary objective was to explore the impact of known drivers of Internet activity in our GT data. In this analysis, the RSV trends for each cancer were temporally compared to events previously reported to increase search activity, including cancer awareness months, celebrity events, and heavily covered news stories [19-22]. In this analysis, RSV was determined by setting 100 at the time period with greatest activity for the United States as a whole. Cancer awareness months for each of the 6 cancers of interest are October (breast), September (prostate), November (lung), March (colon), and September (uterine and leukemia). For other noticeable spikes in RSV, Google searches were performed in a 2-step process first using the cancer and date range to identify news stories for that timeframe: “lung cancer March 2010.” If that search was not productive, a second search with the term “celebrity” was added: “lung cancer March 2010 celebrity.”

### Selection of Study Population

Our study focused on GT annual RSV for cancer by state for the years 2004 through 2013, selected because GT starts in 2004 and the most recent cancer incidence data published by the CDC is for the year 2013.

### Statistical Analysis

Our outcome of interest was correlation between RSV and cancer incidence at the state level from 2004 through 2013. For all 6 cancers selected, we obtained annual RSV and cancer incidence for each state and the District of Columbia (up to 510 RSV and 510 incidence values per cancer). We used the Spearman correlation coefficient to examine the association between state-level GT RSV and state-level cancer incidence per 100,000 people for each year during the 10-year study period. Additionally, we examined the association of state-level RSV and state-level cancer incidence per 100,000 people using linear regression with RSV as the independent variable and state-level cancer incidence per 100,000 people as the dependent variable. For our linear regression, we included time in years as a continuous covariate to control for the fact that cancer incidence and RSV changed over the 10-year study period. RSV data were complete except for uterine cancer. Due to low search volumes for uterine cancer in sparsely populated states, RSV was not present in all states for all years, and we excluded 82 total missing values out of 510 potential observations (50 states and District of Columbia for 10 years).

In a secondary analysis, the aggregated (mean) cancer incidence for each state was obtained from the CDC for the years 2009 through 2013. A 5-year window was selected to reflect more recent cancer incidence and Internet search behaviors and use a shorter time period of aggregate data. The states were then ranked by aggregated cancer incidence and grouped into quartiles. The aggregated RSV for each state was then obtained from GT for the same 5-year period (2009 through 2013) and was similarly divided into quartiles. Quartiles were then compared to one another. For uterine cancer, RSV was not present for 10 states, and these states were excluded from analysis. Rank comparison for secondary analysis by rank quartiles was performed from 1 to 43 for uterine cancer and 1 to 50 for all other cancers.

Statistical analysis was conducted using Stata 14.2 (StataCorp LLC). A 2-sided *P*<.05 was considered statistically significant for all tests.

## Results

### Incidence and Google Search Volume

**Figure 1:**
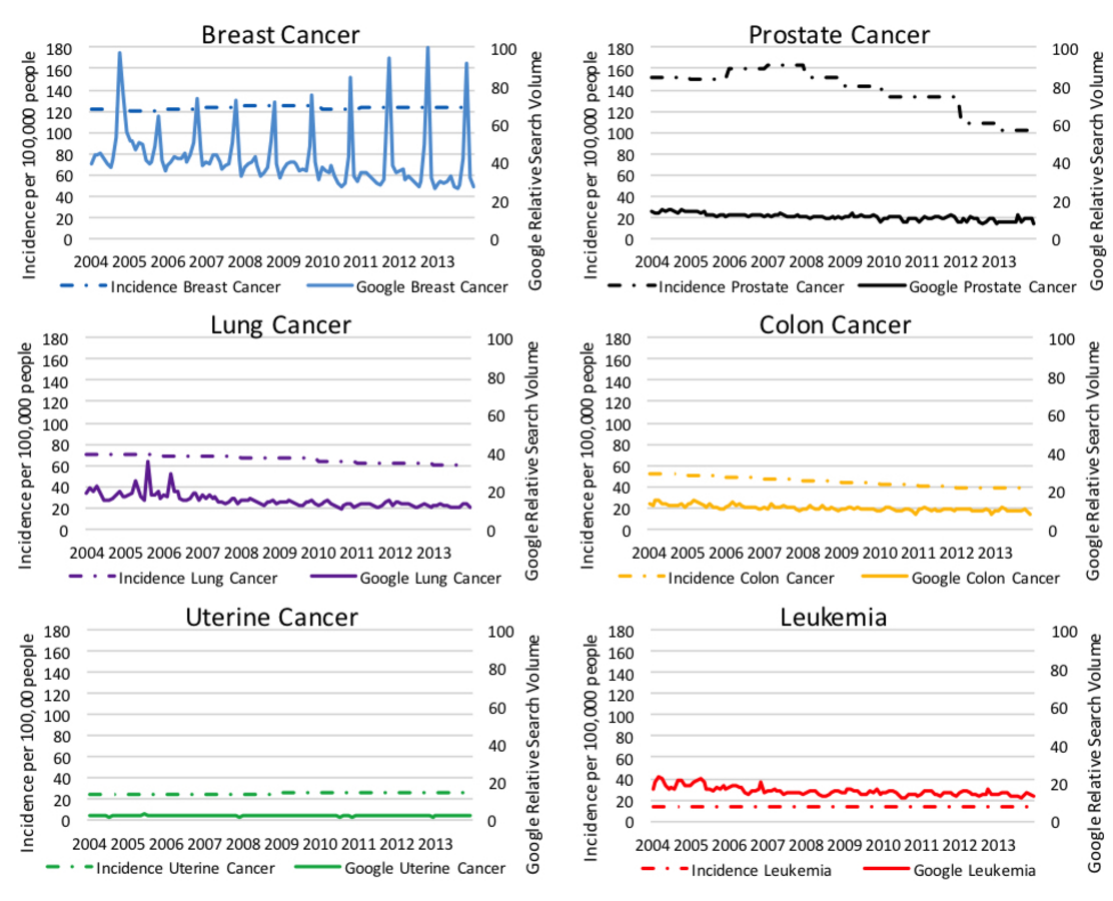
Relative incidence versus Google relative search volume for common cancers 2004-2013.

We examined state aggregate cancer incidence using CDC data and compared this with RSV in 2004 through 2013 (Multimedia Appendix 1). The median aggregate incidence per 100,000 people for each cancer from 2004 through 2013 was as follows: breast 123.5, prostate 142.3, lung 66.7, colon 44.8, and uterine cancer 25.1 and leukemia 13.5. Regarding cancer incidence, prostate and colon cancer decreased over the study period (Figure 1). Cancer incidence for the other cancers studied was relatively constant. The median aggregate RSV over the study period for each cancer was breast 66.7, prostate 63.2, lung 62.5, colon 66.9, and uterine cancers 53.2 and leukemia 70.5. These RSV represent the searches for each cancer term compared between states. In addition, we compared the RSV of the cancer terms to each other during the 2004 through 2013 study period for the United States as a whole, with breast cancer being the most searched (Figure 1).

### Correlation and Regression Comparing State-Level Cancer Incidence and Google Trends Relative Search Volume

Using the Spearman correlation, state-level RSV was significantly correlated to state-level cancer incidence for breast (*r*=.18, *P*=.001), prostate (*r*=–.27, *P*<.001), lung (*r*=.33, *P*<.001), and uterine cancers (*r*=.39, *P*<.001) and leukemia (*r*=.13, *P*=.003) but not for colon cancer (*r*=–.02, *P*=.66) (Table 1). Linear regression demonstrated consistent results for the positive and negative correlations seen using the Spearman correlation. After adding time to our regression to account for changes in cancer incidence and RSV, the coefficients for all cancers were positive with a range of .02 (95% CI 0.01 to 0.03) for leukemia to .39 (95% CI 0.33 to 0.46) for lung cancer (Table 1). Prostate cancer was the only cancer not to have a statistically significant positive association (*P*=.38, 95% CI –0.08 to 0.22). The coefficients of determination ranged from a low of .03 for leukemia to a high of .56 for colon cancer.

**Table 1:** Correlation and linear regression for state-level Google relative search volume and cancer incidence from 2004 through 2013.

CDC^a^ cancer term	Google cancer term	Correlation test		Linear regression with year included		
		Spearman *r*_s_	Spearman *P* value	Coefficient (95% CI)	*P* value	*R*^2^
Breast	Breast cancer	.18	.001	.12 (0.06 to 0.19)	<.001	.05
Prostate	Prostate cancer	–.27	<.001	.07 (–0.08 to 0.22)	.38	.47
Lung and bronchus	Lung cancer	.33	<.001	.39 (0.33 to 0.46)	<.001	.28
Colon and rectum	Colon cancer	–.02	.67	.14 (0.11 to 0.17)	<.001	.56
Corpus and uterus, NOS^b^	Uterine cancer	.40	<.001	.09 (0.07 to 0.12)	<.001	.16
Leukemias	Leukemia	.13	.003	.02 (0.01 to 0.03)	.001	.03

^a^CDC: Centers for Disease Control and Prevention.

^b^NOS: not otherwise specified.

**Figure 2:**
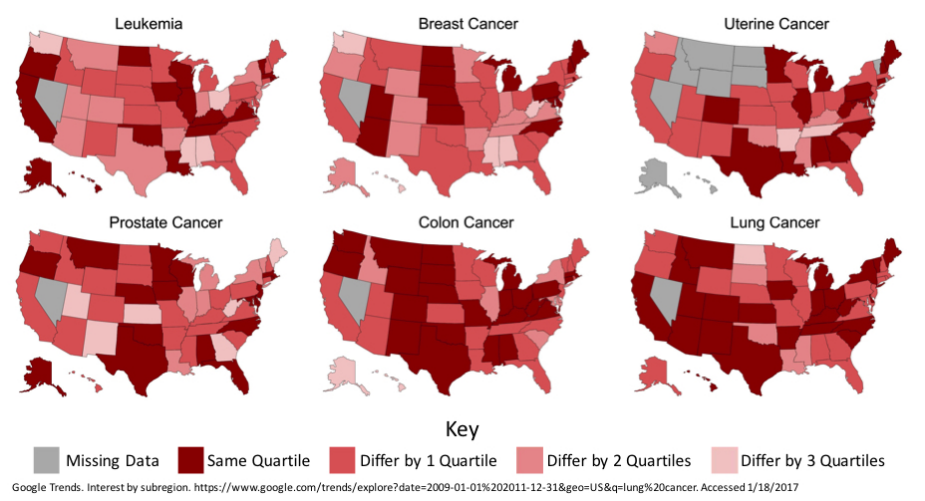
State cancer incidence rank and Google relative search volume (RSV) rank for common cancers by quartile 2009-2013. States whose rank-based quartile was the same for both cancer incidence and RSV are shown in dark red. Progressively lighter shades indicate greater difference in rank of cancer incidence and RSV (quartile difference ranged from 0-3). States shown in gray had missing data and were excluded.

### Comparing Cancer Incidence Rank and RSV Rank by Quartile

The states were ranked in order based on their cancer incidence and RSV from 2009 through 2013 and grouped into quartiles. Figure 2 depicts a map of the United States highlighting the degree of agreement by quartile for a state’s average cancer incidence and average RSV. When grouped by quartile, some cancers demonstrate a higher agreement of state cancer incidence rank and state RSV rank than others.

### Cancer Awareness Months and News Events

Search spikes were common for breast cancer and less so for the other cancers. Breast cancer has a large increase in searches during the month of October each year (Figure 1). These annual spikes represent increases in search volume that are more than double the baseline search volume for breast cancer and are temporally associated with breast cancer awareness month. Excluding breast cancer awareness month from our time-adjusted regression raised the coefficient of determination from .05 to .07. No other cancer had a spike in activity during its awareness month.

**Figure 3:**
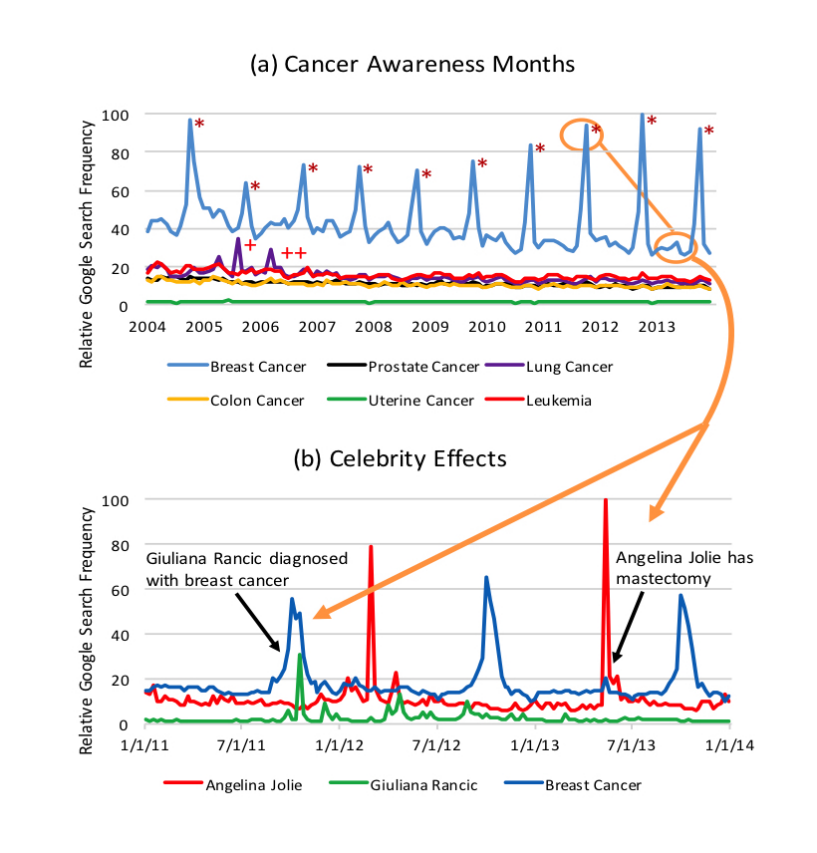
Temporal relationship of Google Trend data to public events. (A) Trends in relative search volume (RSV) for all 6 cancers of interest from 2004-2013. *Breast cancer awareness month. +Death of Peter Jennings. ++Death of Dana Reeve. (B) Independent RSV trends for Angelina Jolie, Guiliana Rancic, and breast cancer.

Lung cancer was the only other cancer to have at least 1 RSV spike that was double its baseline RSV. The 2 RSV spikes for lung cancer occurred in August 2005 and March 2006, which were temporally associated with the deaths of public figures (Peter Jennings and Dana Reeve) from lung cancer (Figure 3). For breast cancer, spikes in RSV were temporally noted with Angelina Jolie’s mastectomy and Guiliana Rancic’s public announcement of breast cancer (Figure 3).

## Discussion

### Principal Findings

In this study, we examined the association of Google search activity with cancer incidence over time across the United States. Our results demonstrate a significant association between Google search activity and incidence of 5 of the 6 common cancers at the state level. The strength of association between RSV and cancer incidence varied among the cancers studied. Conclusions drawn from online search volume about one type of cancer may not be able to be generalized to other types of cancer. We see a similar pattern of limited generalizability in studies of the relationship of online search activity to disease incidence for subtypes of influenza where searches for H1N1 were different compared to other types of influenza [15-16] and, in a prior study within oncology, incidence positively correlated to Google search volume for 5 of 8 cancers studied [23]. Similarly, we found a positive correlation for 4 of 6 cancers, and the strength of positive correlation for all 6 improved when time was added to a regression model, with only prostate cancer failing to reach significance.

Incorporating time into the model had the greatest impact for colon and prostate cancer due to their declining incidence during the study period; both changed from a negative Spearman correlation coefficient to a positive regression coefficient (Table 1). Because the RSVs are set to 100 for each year in the analysis, a declining underlying population incidence effectively changes what 100 represents in a given year. Caution should be taken when interpreting online data for diseases with an incidence that changes over time.

Other lessons applicable to Internet surveillance research involve the cautionary tale of the GFT. After initial success, it proved to overestimate flu incidence as a result of mismatching correlated terms, different media coverage levels between flu seasons, and lack of algorithm transparency [17]. Our work differs in that it does not use correlated search terms, and cancer has a different online search profile compared to infectious diseases. For example, cancer is typically not searched for on a seasonal basis, and media coverage of cancer is likely to be different than the flu. We attempt to account for the media coverage by examining searches relative to awareness months and explicitly searching for news stories when the RSV data showed an unexpected rise.

Temporal associations are present for news stories and popular culture. The breast cancer RSV curve has spikes in activity that are temporally related to reports of Angelina Jolie’s mastectomy and Guiliana Rancic’s public announcement of breast cancer (Figure 3). The “Jolie Effect” has been described in increases in breast cancer susceptibility gene (BRCA) testing following her public disclosure [25] and more websites addressing common themes regarding care for patients with BRCA mutations after her public announcement [26]. Knowing the news pulse for specific stories such as Ms. Jolie’s mastectomy in response to her BRCA status offers opportunities for targeted medical messaging by the public health community that overlaps with an increase in public interest in that topic.

Other celebrity events temporarily associated with RSV spikes include the deaths of Peter Jennings and Dana Reeve from lung cancer. If search data are to be considered for the purpose of surveillance, current events and drivers of Internet activity must be taken into account, as these drivers may obscure the relationship between searches performed in response to direct impact on individuals and those driven by news or public information. Further work is needed to clarify these potential confounders of the relationship between cancer incidence and search activity and improve the utility of Google search data.

Despite factors other than cancer incidence driving searches, we found online search activity mirrors cancer incidence. Cancer clusters with unusually high incidence have been reported [27], and online adjunctive surveillance may have been useful in detecting these hot spots. Online search activity is less likely to be relevant in trending the national incidence, which has well-established surveillance and reporting methods. With appropriate transparency in trend algorithms, further work, and appropriate input from the scientific community, meaningful public health initiatives and adjunctive cancer surveillance methods could be achieved.

In addition to detecting signals about cancer incidence, search data are exquisitely good at reflecting people’s interest at the population level. Our data add to the literature supporting news coverage, and cancer awareness campaigns can register with the general public and drive online activity. It remains unclear why breast cancer is the only cancer studied that demonstrated a significant increase in its RSV during its cancer awareness month. The granularity of RSV data allows for assessment of the impact of public health campaigns and public awareness at the national, state, and metropolitan area levels. The most practical current application for online surveillance may be assessing changes in public engagement after an event or campaign to increase public knowledge about a cancer topic.

Online data provide information about which cancers could be the best targets for digital outreach. For example, prostate cancer was the only cancer studied without a significant association between RSV and incidence and may be a poor choice for online interventions. One explanation for the lack of significant association could be that the population with prostate cancer tends to be elderly men. According to the US Census Bureau, in 2010 only 55% of people aged 65 years and older had Internet access in their homes [28]. It is possible that association with RSV for prostate cancer will become significant as the current population integrated into Internet use ages. Understanding the relevance of online searches for a given cancer could inform patient-centered approaches to distribute information for many aspects of cancer care including trial recruitment, screening practices, and care options.

Finally, establishing a link between online search activity and cancer incidence is of use to those interested in mining the Internet and social mediome for medically relevant information. Internet searches provide data that indicate what people want to know and when they want to know it. Linking cancer search volume to incidence provides validity to work examining correlations between cancer terms and other aspects of cancer care such as treatment side effects.

### Limitations

This study has some limitations. It did not include searches done through alternative search engines such as Yahoo or Bing. The algorithm employed by Google to determine the RSV was not published and could contain systematic errors. GT reported data at the state level and for major metropolitan areas. It was less well suited for rural areas and uncommon topics. Additionally, GT cannot link to a specific user, and data were only available at the population level. We cannot therefore control for confounders that may impact search activity and cancer incidence such as race, ethnicity, smoking status, socioeconomic status, and level of education.

### Conclusion

Our 3 key findings are cancer incidence is correlated with Google search volume at the state level, different cancers demonstrate unique Google search patterns, and search patterns are influenced by public events such as cancer awareness months and news coverage of celebrity experiences with cancer. Online searches reflect public awareness, and advancing understanding of online search patterns could augment traditional epidemiologic surveillance, provide opportunities for targeted patient engagement, and allow public information campaigns to be evaluated in ways previously unable to be measured.

## Appendix

Multimedia Appendix 1 Average incidence per 100,000 people and average relative search volume by state from 2004-2013.

## Abbreviations

BRCA: breast cancer susceptibility gene
CDC: Centers for Disease Control and Prevention
GFT: Google Flu Tracker
GT: Google Trends
NCI: National Cancer Institute
RSV: relative search volume
SEER: Surveillance, Epidemiology, and End Results
