# Supplementary material for: Relationship Between State-Level Google Online Search Volume and Cancer Incidence in the United States: Retrospective Study
Source: J Med Internet Res. 2018 Jan 8;20(1):e6. doi: 10.2196/jmir.8870 (PMC5778251; doi:10.2196/jmir.8870)
Supplement: Multimedia Appendix 1 [file jmir_v20i1e6_app1.pdf]

Multimedia Appendix 1. Average incidence per 100,000 people and average relative search volume (RSV) by state from 2004-2013

State	Breast Cancer		Prostate Cancer		Lung Cancer		Colon Cancer		Uterine Cancer		Leukemia	
	IR	RSV	IR	RSV	IR	RSV	IR	RSV	IR	RSV	IR	RSV
Alabama	118.3	70.7	149.5	71.5	73.9	64.4	47.3	77.3	18.4	50.5	11.8	73.7
Alaska	126.9	62.2	123.1	67.0	69.0	55.8	47.6	68.9	24.0	--	12.7	59.7
Arizona	110.4	58.8	105.4	61.3	54.2	51.5	37.0	59.8	20.2	52.1	11.0	59.6
Arkansas	110.6	66.7	143.3	75.1	79.5	65.9	45.8	77.9	19.8	58.8	11.5	71.0
California	122.0	49.8	134.2	50.9	50.1	43.7	41.5	51.9	23.2	37.0	12.6	50.5
Colorado	123.9	55.8	144.2	57.4	48.2	51.2	38.7	59.0	21.0	49.7	13.9	56.7
Connecticut	136.8	70.7	147.2	67.4	65.5	68.1	44.6	66.6	29.1	66.1	14.1	70.5
Delaware	128.1	95.7	167.8	83.6	76.2	85.6	44.6	78.1	28.3	--	13.5	79.8
D. of Columbia	139.5	65.9	182.0	64.4	60.4	53.7	47.1	62.2	28.4	48.3	9.7	60.4
Florida	115.4	58.9	126.0	61.1	66.7	53.2	42.1	59.9	22.1	46.1	13.2	57.1
Georgia	122.5	65.2	155.3	59.7	70.5	55.8	44.8	64.2	19.8	46.9	12.8	60.3
Hawaii	126.6	54.5	113.7	61.2	50.3	51.9	46.4	65.3	29.0	--	11.3	57.9
Idaho	117.9	55.5	151.4	65.3	54.4	49.9	39.8	65.9	23.4	--	15.6	70.9
Illinois	126.7	66.0	144.8	59.4	70.3	62.2	49.9	65.3	27.9	53.1	13.5	63.5
Indiana	118.7	71.7	119.4	63.2	77.0	72.0	47.8	72.6	26.4	64.6	13.3	80.9
Iowa	122.4	70.7	130.8	62.2	66.9	69.6	49.6	76.9	29.9	63.8	15.5	85.2
Kansas	123.5	61.9	147.9	57.3	64.9	54.9	45.6	65.8	24.0	52.3	14.9	73.7
Kentucky	121.5	70.7	130.2	66.6	99.0	84.0	53.8	80.4	24.3	60.9	14.6	75.8
Louisiana	120.1	64.9	161.7	62.3	74.9	60.7	51.3	66.9	17.9	45.6	12.8	66.2
Maine	127.3	72.3	135.5	68.8	77.2	74.4	45.9	73.5	30.7	78.5	15.9	72.8
Maryland	127.8	75.8	147.4	77.6	63.6	67.9	41.6	72.0	24.4	56.8	11.7	74.1
Massachusetts	135.4	70.1	145.6	64.7	69.4	64.7	44.1	61.8	29.8	58.0	12.9	67.1
Michigan	122.6	66.1	154.7	61.1	71.1	62.5	43.9	62.2	28.4	58.4	14.0	68.0
Minnesota	128.4	65.5	157.2	63.2	56.4	63.3	43.3	70.7	28.3	57.2	16.0	69.7
Mississippi	114.4	73.3	158.0	69.4	78.8	69.8	51.8	75.5	19.4	50.0	11.9	76.6
Missouri	123.5	64.4	120.8	60.1	78.2	62.4	47.1	67.7	25.2	53.2	13.1	71.5
Montana	122.6	61.6	149.0	80.8	61.8	59.6	43.2	66.2	24.8	--	15.2	74.7
Nebraska	122.9	68.7	138.4	62.3	61.8	68.8	49.6	78.1	26.4	55.2	14.3	78.8
Nevada	78.4	50.3	95.2	56.9	48.8	48.7	31.7	54.3	12.8	36.2	9.6	54.1
New Hampshire	135.9	74.0	147.3	66.2	70.4	70.7	43.2	66.4	31.3	73.3	14.4	65.3
New Jersey	130.8	68.4	160.9	66.3	62.1	62.5	47.5	64.3	30.1	59.0	14.3	63.0
New Mexico	111.5	69.5	122.6	77.2	44.5	54.7	38.1	68.1	21.6	53.0	13.0	72.6
New York	127.5	64.0	158.3	59.6	63.5	59.5	45.6	60.7	30.0	51.1	15.1	62.0
North Carolina	126.4	68.4	146.5	68.2	73.5	65.6	42.8	69.2	22.8	54.6	12.9	69.9
North Dakota	123.2	78.5	149.7	70.0	56.9	79.0	50.4	92.7	25.2	--	15.8	81.8
Ohio	122.1	66.7	134.2	59.9	73.4	65.5	46.3	66.6	27.8	66.9	12.0	74.0
Oklahoma	121.6	59.5	136.3	63.3	76.5	53.9	46.4	69.8	20.7	53.7	14.3	62.9
Oregon	129.6	51.1	130.7	59.8	63.4	48.4	40.7	55.8	25.6	52.6	12.1	57.8
Pennsylvania	127.2	72.8	141.5	67.6	68.4	69.7	48.6	68.8	31.2	66.7	14.0	76.7
Rhode Island	131.8	75.4	137.9	59.4	72.1	72.4	44.8	69.4	30.8	50.6	14.1	76.7
South Carolina	123.9	68.4	150.4	73.1	71.5	70.2	44.2	77.2	21.1	48.4	12.9	71.1
South Dakota	125.3	83.6	142.3	79.8	59.4	78.9	47.2	78.8	26.5	85.7	13.9	85.8
Tennessee	120.1	69.1	135.2	70.1	79.3	66.7	45.5	79.8	20.7	63.4	13.0	73.2
Texas	113.7	56.0	127.7	53.8	61.1	47.7	42.7	59.6	19.8	43.1	13.9	60.6
Utah	112.6	47.9	164.2	56.2	28.9	38.8	34.9	61.0	24.0	51.3	13.7	61.0
Vermont	129.1	72.8	132.9	73.1	69.2	69.0	41.2	78.7	32.2	--	13.3	69.7
Virginia	125.1	54.4	138.6	51.4	65.5	48.8	41.5	59.7	23.1	42.7	11.0	52.5
Washington	133.0	55.2	144.4	57.1	63.1	47.7	40.7	57.8	25.1	49.2	14.8	58.5
West Virginia	113.6	86.4	122.9	79.3	85.9	90.5	51.0	92.6	29.7	71.2	14.2	86.4
Wisconsin	125.7	67.3	139.8	62.7	62.6	64.9	42.8	64.3	28.8	63.2	16.5	74.4
Wyoming	111.2	75.0	139.3	60.0	49.9	61.9	40.9	72.4	20.9	--	12.0	75.0
First Quartile	119.4	60.6	131.9	59.9	60.8	53.8	41.9	62.2	21.3	49.9	12.6	60.8
Median	123.5	66.7	142.3	63.2	66.7	62.5	44.8	66.9	25.1	53.2	13.5	70.5
Third Quartile	127.4	71.2	149.6	69.1	73.5	69.3	47.3	74.5	28.6	62.1	14.3	74.6

IR incidence rate per 100,000 people; RSV relative search volume
